# A Copper Oxide/Zinc Oxide Composite Nano-Surface for Use in a Biosensor

**DOI:** 10.3390/ma12071126

**Published:** 2019-04-06

**Authors:** Lu Cao, Janice Kiely, Martina Piano, Richard Luxton

**Affiliations:** Institute of Bio-Sensing Technology, University of the West of England, Frenchay Campus, Bristol BS16 1QY, UK; Janice.Kiely@uwe.ac.uk (J.K.); Martina.Piano@uwe.ac.uk (M.P.); Richard.Luxton@uwe.ac.uk (R.L.)

**Keywords:** biosensor, impedance, ZnO–CuO, CRP

## Abstract

In this study, biosensors based on zinc oxide–copper oxide composite nano-surfaces were prepared using a simple and inexpensive distributed colloidal technique. Combinations of mixed dispersions with volume ratios of 1:1, 1:2 and 2:1 ZnO:CuO were compared. The uniform nano-crystalline sensor surfaces on polyethylene terephthalate (PET) were analysed using scanning electron microscopy (SEM), Atomic Force Microscopy (AFM) and Raman Spectroscopy. The ZnO–CuO composite biosensor nano-surfaces showed a significantly increased impedimetric signal compared with pure ZnO nanocrystals, and the maximum output was achieved with a volume ratio of 1:2 ZnO/CuO. The antibody capture of C-reactive protein (CRP) on the nano-surfaces was used to demonstrate the enhanced signal generated with increasing amounts of CuO in the nano-surface.

## 1. Introduction

Zinc oxide (ZnO) has been employed for a variety of applications, for example, in antibacterial agents [1], photo-catalysts [2], nanogenerators [3] and gas sensors [4,5]. Because of the key properties of ZnO nanoparticles, i.e., high electron mobility, good chemical stability, low toxicity and biological compatibility [6], ZnO biosensors are also widely used in applications for detection of small molecules such as glucose [7], cholesterol [8] and DNA [9].

Enhancements of material properties have been demonstrated by incorporating other materials or elements within a ZnO matrix. For example, the implantation of iron oxide (Fe${}_{3}$O${}_{4}$) in ZnO nanosheets decreases the charge transfer resistance and also ensures that the nanosheets are photostable and reusable, and thus, suitable for application as photocatalysts [2]. In order to enhance the electrocatalytic properties of a modified electrode, carbon xerogel-zinc oxide composites were synthesized by the sol-gel method, contributing to good material conductivity [10].

The addition of copper oxide (CuO) to ZnO has been shown to improve sensitivity by enhancing the redox property and electron transfer. ZnO surfaces have been created by an electro-spinning method followed by hydrothermal treatment, with CuO subsequently being added by a wet method [4]. These “CuO-decorated ZnO hierarchical nanostructures” were employed to enhance the performance of a Hydrogen Sulfide (H${}_{2}$S) gas-sensor. Furthermore, a novel free-standing ZnO–CuO composite film, which was fabricated by a modified hydrothermal method, in order to remove the need for a substrate [5], formed the basis for a gas sensor which showed a good improvement in response for sensing carbon monoxide (CO) gas compared with pure ZnO nanowires [5]. Finally, a ZnO–CuO composite matrix was fabricated on an ITO (Indium tin oxide) coated corning glass substrate by pulsed laser deposition [11]. The inclusion of CuO resulted in excellent redox properties, and consequently, in the absence of any external mediator, good oxidation and reduction peaks were visible on a cyclic voltammogram. The improvement in properties in these composite nanoparticles has been related to the decrease in the band gap energy. In fact, it has been shown that the higher the concentration of CuO in a ZnO–CuO composite, the smaller the band gap [1].

However, for all of these examples plus others in the literature, the formation of ZnO/CuO composite nanoparticles/nano-surfaces requires complex processes, typically involving high temperature and long time periods. For example, ZnO nanowires have been grown by thermal evaporation and CuO films have been synthesized by thermal oxidation (at 400 ${}^{\circ }$C for 12 h in 80% oxygen/20% argon atmosphere); the Cu films initially being produced by a radio frequency (RF) sputtering technique [12]. Methods described for fabricating CuO–ZnO nanocomposites are also complex. For example, Widiarti et al. [1] added copper(II) sulphate pentahydrate (CuSO${}_{4}$·5H${}_{2}$O) to citric acid stirring until homogenous and the precursor solution made by ethylene glycol mixed with zinc(II) acetate dihydrate (Zn(CH${}_{3}$COO)${}_{2}$·2H${}_{2}$O), deionized water and citric acid by vigorous stirring for an hour. The solution was added by sodium hydroxide (NaOH) drop by drop stirring for one hour and kept for 48 h for aging. The gel was washed and dried in oven to constant weight and then calcined at 500 ${}^{\circ }$C for 4 h [1].

The study presented here investigated mixtures of ZnO and CuO nanoparticles to fabricate nanocrystal surfaces for the immobilisation of anti-CRP antibodies. An enzyme labelled antibody was used to assess protein uptake on the nano-surfaces. The fabrication of the biosensor nano-surfaces used a simple and inexpensive colloidal dispersion technique. ZnO nanocrystals were deposited on PET, coated with anti-CRP antibodies and then sandwiched between two glass coverslips for the detection of CRP. The binding of CRP to the antibody on the biosensor surface was detected and quantified using impedance spectroscopy. Fabrication of the ZnO–CuO nano-surfaces was easy to fabricate, with a process that is compatible with large scale manufacture.

C-reactive protein (CRP) was chosen as the model target for the biosensor as it is an important marker of inflammation and can also predict myocardial infarction and sudden cardiac death [13]. A rapid test which can be used in a doctors surgery of clinic will allow the assessment of patients during their consultation without the need to send a blood sample to a centralise laboratory for testing. The use of paper based lateral flow or paper based microfluidic assays have been described [14] highlighting a point-of-care application for the measurement of CRP.

## 2. Materials and Methods

### 2.1. Surface Preparation

The sum of 0.15 g ZnO nanoparticles (99.9+%, 80–200 nm from US Research Nanomaterials Inc., Houston, TX, USA) was added to 15 mL double deionized water and 0.1 g CuO nanoparticles (99.5+%, width 10–30 nm, Length: 200–800 nm, Long fibrous shape from US Research Nanomaterials Inc.) was added to 10 mL double deionized water. The suspensions of both 1% ZnO and 1% CuO were stirred at room temperature for 1 h. Five different nano-surfaces were produced as follows: 0% CuO (ZnO only), 33% CuO (2:1 ZnO:CuO via volume ratio), 50% CuO (1:1 ZnO:CuO), 67% (1:2 ZnO:CuO) and 100% CuO (CuO only). 1.5 mL aliquots of each preparation (prepared by volume ratios) were ultra-sonicated for 7 periods of 20 s, at 4 min intervals using an exponential microprobe (Soniprep 150) at 30 watts. 200 μL of each suspension were dropped on to clean polyethylene terephthalate (PET) substrates (20 mm × 20 mm) separately to make ZnO/CuO nano-surfaces. Finally, they were dried in an oven at 65 ${}^{\circ }$C for 80 min and cooled down to room temperature, then stored in a dry atmosphere with silica gel for up to 2 days [15].

### 2.2. CRP Sensor Fabrication and Test

The sensing area (10 mm × 4 mm) of each ZnO–CuO nano-surface on PET was defined by tape. Subsequently, 40 μL (100 ng or 200 ng) of monoclonal mouse anti-human C-reactive protein from HyTest Ltd. (Turku, Finland) was added to the surface. Phosphate Buffered Saline (PBS, pH 7.3 ± 0.2 at 25 ${}^{\circ }$C) was purchased from OXOID Microbiology products (Fisher Scientific, Loughborough, Leicestershire, UK). Anti-CRP antibodies were stored and diluted in 0.025 M PBS with pH 7.40. The biosensor was then dried in a desiccator with silica gel at 4 ${}^{\circ }$C overnight for 18 h. The nano-surface biosensors were then aligned above a pair of D-shape electrodes to perform impedance measurements.

A Cypher Instruments C60 Impedance-Amplitude-Phase Analyser (Cypher Instruments, UK) was used to measure the impedance of the nano-crystal surfaces. The frequency was scanned from 10 Hz to 4 MHz at a voltage of 2 Vpp with a DC offset of 0.9 mV, with 300 test points. The impedance plots were analysed by Cypher Graph V1.21.0, Impedance Amplitude and Phase Analyser graphing application software. Impedance spectra of the ZnO–CuO nano-surfaces were acquired in triplicate for each of the following types of surface: 1% ZnO and 1:1, 1:2 and 2:1 volume ratios of 1% ZnO and 1% CuO suspensions.

The impedance amplitude and phase were measured after 10 min incubation time following the addition of 40 μL of different concentrations of human C-reactive protein (CRP) from HyTest Ltd. and sealing the sensing region to form a chamber using cover slip. The specific concentrations of CRP were as follows: 0, 1, 5, 10 and 100 ng/mL diluted in 0.025 M PBS with pH 7.40. To demonstrate specificity of the anti-CRP antibody, a negative control consisting of 125 ng/mL cortisol (Co-pharma) was added to the sensor surface composed of 1% ZnO (n = 2) with 200 ng anti-CRP antibody.

**Figure 1:**
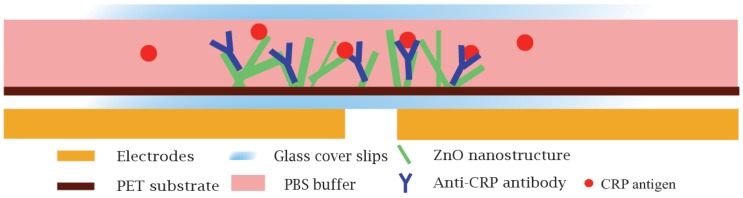
The cross sectional illustration of biosensor.

The cross section of one of the biosensor (ZnO only) is illustrated in Figure 1. This shows the sensing area with ZnO nanocrystals, on PET and dry anti-CRP antibodies for the detection of CRP sandwiched between two coverslips.

The sensor response was plotted using readings taken at a frequency of 138 Hz. This frequency was selected as in previous work it demonstrated maximum changes in response to addition of a target analyte [15]. This observation concurred with Jacobs et al., who found that the electrical double layer is greater at frequencies below 1000 Hz and the most significant changes occurred around 100 Hz in their experiment to measure the protein troponin-T on a ZnO surface [16]. Impedance values were compared with both 100 ng and 200 ng anti-CRP capture antibody. The phase change was defined as the difference of the phase value after adding CRP, after 10 min incubation, and the control. In order to plot logarithmic concentrations of CRP, the measurement of PBS buffer only with no CRP (the blank), was defined as 0.1 ng/mL CRP (rather than 0 ng/mL).

### 2.3. Characterization

The morphology of pure ZnO and mixed ZnO–CuO composite nano-surfaces were analysed by the Quanta 200 Scanning Electron Microscope (Thermo Fisher Scientific, Hillsboro, OR, USA). The samples were coated with a thin layer of Au prior to analysis.

AFM was used to examine the topology of the different nano-surfaces using Bruker Innova Atomic Force Microscope (Bruker UK limited, Conventry, UK) with an antimony (n) doped silicon tip. Each AFM image was analysed using the NanoScope Analysis software (version 1.8). Image surface areas were compared within a 3 μm by 3 μm scan area. Calculation of the total surface area and mean roughness (Ra) within the scanned region provided a method of comparing the roughness of the various nano-surfaces.

Raman spectroscopy was used to analyse the chemical compositions of the pure 1% ZnO and mixed 1% ZnO–CuO (1:2) nano-surfaces. An XploRA Raman spectrometer from Horiba (HORIBA UK Limited, Northampton, UK), equipped with a confocal microscope, was used. The Raman signals were collected in a range of 0–3500 cm${}^{-1}$ using a 785 nm red laser excitation. The laser beam was focused on the sample using objective magnification of 50×.

A Kruskal-Wallis test was used to analyse the data and level of significance was defined as *p* ≤ 0.05.

### 2.4. Surface Uptake of Antibody

To understand how ZnO and CuO captured protein on their surfaces an antibody-enzyme conjugate was used in place of the anti-CRP. Here, 75 μL anti-mouse Ig-HRP (2.5 ng/μL) from Bio-Rad Antibodies Inc. (Kidlington, UK) was dropped on each surface within the sensing area and dried in a desiccator with silica gel at 4 ${}^{\circ }$C overnight for 18 h. Following a thorough wash, to remove unbound protein, with 0.05 M PBS, 40 μL 3,3’,5,5’-Tetramethylbenzidine (TMB) was added on the surface for 10 min after which 50 μL stop TMB solution was added. The colour intensity of each test was read at 450 nm using a microplate reader (EZ Read 400, Biochrom Ltd., Cambridge, UK). The relative amounts of surface bound antibody were defined by the optical density of the tests of the different sensor surfaces.

Data were presented with one blank sample on each nano-surface, without anti-mouse Ig-HRP, as a control and two test samples (with antibody) on each nano-surface.

## 3. Results and Discussion

### 3.1. Morphological Study

**Figure 2:**
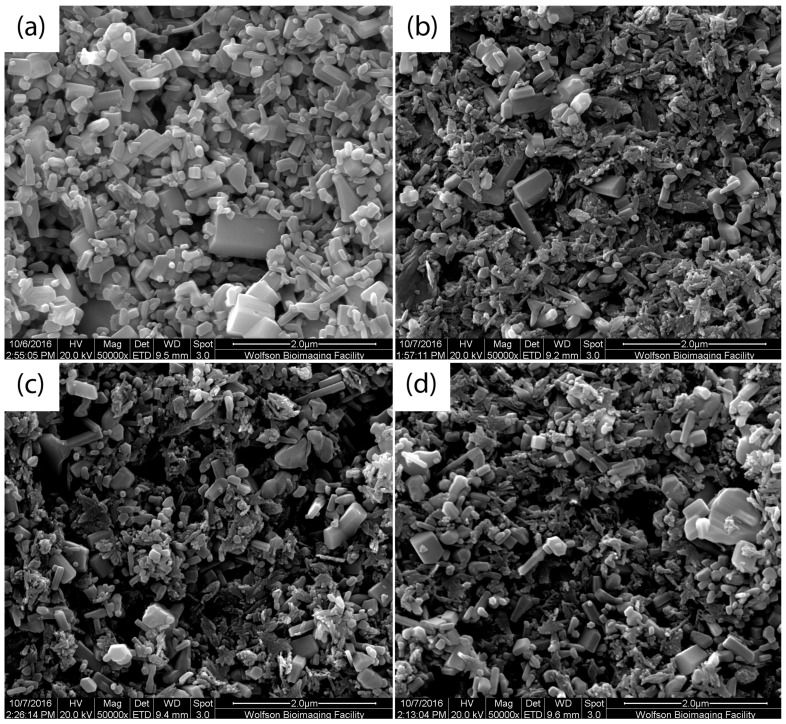
SEM images of nano-surfaces: (**a**) 1% pure ZnO (**b**) 1% ZnO and CuO suspensions with the ratio of 1:2 (**c**) 1:1 (**d**) 2:1.

Figure 2 shows SEM images of nano-surfaces of 1% ZnO and different combinations of 1% ZnO and 1% CuO suspension after ultra-sonication with the ratio of 2:1, 1:1 and 1:2 ZnO:CuO. Figure 2a clearly shows the columnar wurtzite structure of ZnO nanoparticles with a number of voids within this structure. Figure 2b–d show the effects of adding increasing amounts of CuO nanoparticle flakes to the nano-surface (67% CuO, 50% CuO and 33% CuO respectively). It can be seen that the CuO plates pack into the spaces within the ZnO structure having the effect of creating a smother and less “pitted” surface.

**Figure 3:**
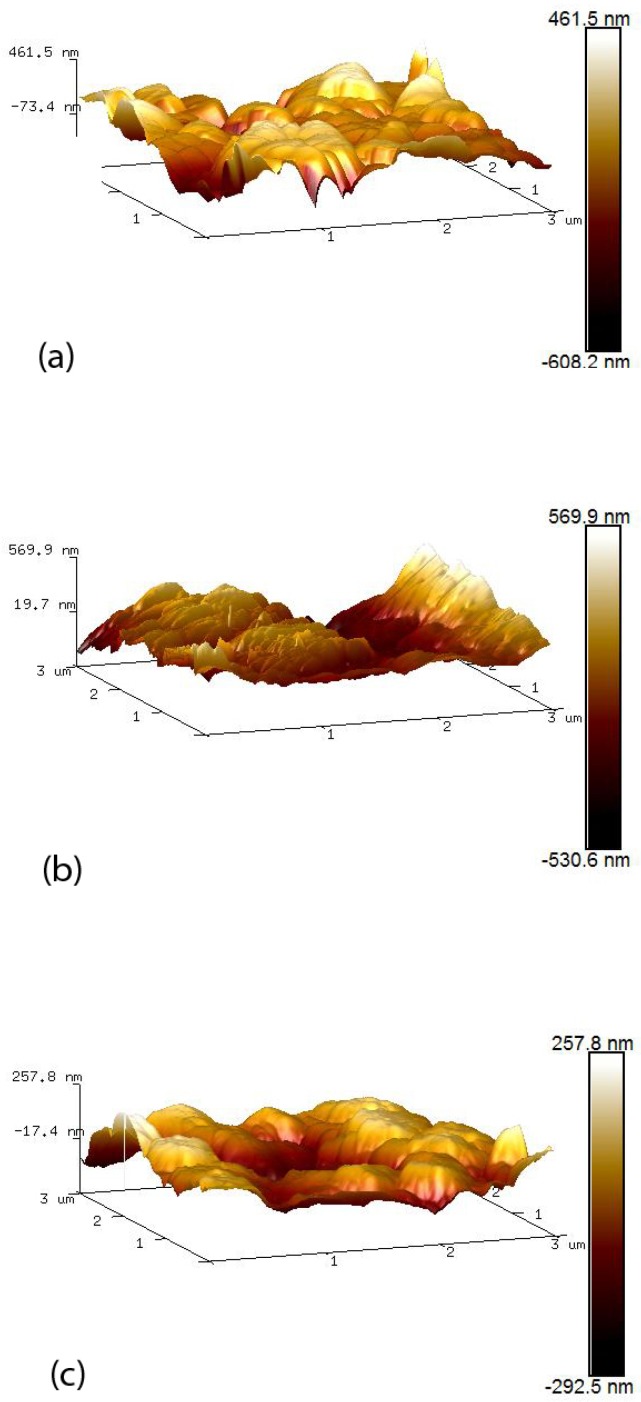
AFM 3D images of 3 μm by 3 μm area of the nano-surfaces: (**a**) 1% pure ZnO; (**b**) 1% pure CuO; (**c**) 1% ZnO and CuO suspensions with the ratio of 1:2.

Figure 3 shows 3D surface model AFM images (areas of 3 μm by 3 μm) of pure ZnO and CuO as well as a mixed nano-surface. The images demonstrate that the CuO nanoparticles are distributed evenly across the ZnO nano-surfaces for the mixed ZnO–CuO surface of Figure 3c. Consequently, the smaller CuO nanoparticles tend to smooth the surface of the mixed nanostructure relative to the ZnO nano-surface. Table 1 shows both surface area (μm${}^{2}$) and mean roughness (Ra) of nano-surfaces. Ra gives a good general description of the height variations among the nano-surfaces. From Table 1, the ZnO nano-surface shows largest surface area. Although CuO nanoparticles are smaller than ZnO nanoparticles they exist as flake like structures with a very high surface area, reflected in the large roughness measured. Interestingly, the mixed nano-surface had both the smallest surface area and roughness. Inspection of the SEM images suggests that the CuO nanoparticles fill deep voids between ZnO nanoparticles and that the CuO nanocrystals appear to lay flat on the ZnO nanocrystal. This is due to the fact that ZnO (n-type) and CuO (p-type) nanoparticles have opposite charges resulting in attraction between the two materials.

### 3.2. Raman Spectroscopy Analysis of the Nano-Surfaces

**Figure 4:**
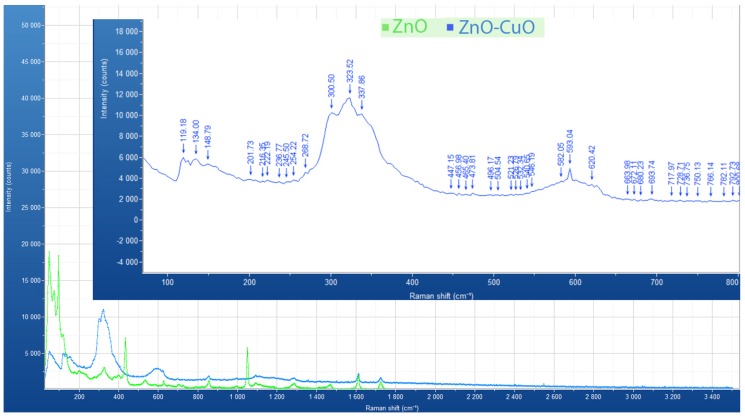
Raman spectra of pure ZnO (green line) and ZnO/CuO composite (blue line) nano-surfaces at room temperature. Conditions of recording the Raman Spectrum of the ZnO nano-surface: time acquisition 50 s, wavelength 785 nm. Conditions of recording the Raman Spectrum of mixed ZnO–CuO nano-surface: time acquisition 300 s, wavelength 785 nm. The inset shows a magnified view of the 100–800 cm${}^{-1}$ section of the Raman spectra of ZnO/CuO composite (blue line) nano-surfaces.

Figure 4 (green) shows bands at 90, 324, 435, 532, 582 and 1050 cm${}^{-1}$. This strongly indicates that the ZnO nanocrystals cover the surface of the PET. 90 cm${}^{-1}$ and 435 cm${}^{-1}$ prove the phonon modes E${}_{2}$ (low) and E${}_{2}$ (high) of ZnO wurtzite phase [11]. From the magnified region of ZnO–CuO composite spectra, it is shown that there are two obvious peaks at 270–340 cm${}^{-1}$ and 580–630 cm${}^{-1}$. Rashad et al. [17] showed three Raman peaks at 282, 330, and 616 cm${}^{-1}$ on pure CuO nanopartciles with 10 nm ± 2 nm particle size measured in TEM images. Wang et al. [5] found an additional two peaks on the Raman spectra of ZnO–CuO composite at 286 and 627 cm${}^{-1}$ representing crystalline CuO. Batra et al. [11] also observed the onset of well-defined phonon peaks at 216 cm${}^{-1}$ and 625 cm${}^{-1}$ which were attributed to Ag and Bg modes according to the vibrations of oxygen atoms in the CuO matrix. Therefore, the results of Raman spectroscopy in this study prove the formation of ZnO–CuO composite nano-surface due to the coexistence of Raman modes of ZnO and CuO.

### 3.3. Antibody Capture on ZnO–CuO Nano-Surfaces

To understand the relative uptake of protein on the ZnO and CuO nano-surfaces and mixtures, a fixed amount of antibody, tagged with an enzyme, was added to the different nano-surfaces and detected through the generation of a coloured product by the enzyme tag. The colour generated gave a direct indication to the amount of antibody on the surfaces. The absorbance values of the coloured product are given in Table 2, the controls all had absorbance values less than 0.06. There was no significant difference between the amount of antibody bound to ZnO or CuO, but some variability was seen with mixtures of ZnO and CuO. The results indicated that 1:1 ratio of ZnO:CuO had less antibody binding that the pure metal oxides and the 2:1 mixture demonstrated greater binding of antibody. Although absorbance values associated with the 1:2 ZnO:CuO surface was higher than both pure ZnO and CuO and higher than the 1:1 mixture, statistical significance was not obtained. Proteins have multiple positive and negative charges on the surface and immobilisation to the nanocrystal surface is largely due to electrostatic interactions. Pure ZnO and CuO interact with proteins only through negative charges or only positive charges, respectively. Whereas mixtures of ZnO and CuO will interact with proteins through both positive and negative charges on the surface immobilising greater numbers of protein molecules as seen with the 2:1 and 1:2 mixtures. The fact that the 1:1 mixture showed significantly less binding than the pure metal oxide surfaces could be a result of the ZnO and CuO cancelling out some charges thereby reducing the numbers of charges available for interacting with protein molecules. Although there were differences in the physical surface area between the pure metal oxides and the mixtures this was not reflected in the amount of protein that bound to the surfaces. This could be due to the fact that the amount of antibody-enzyme complex did not saturate all the binding sites available on the surfaces.

### 3.4. Detection of CRP on Different ZnO–CuO Composite Nano-Surfaces

The isoelectronic point (pI) of ZnO (9.5) and CuO (8.7–10.3) endow the nano-surfaces with positive charges at the pH of the buffer system. Anti-CRP binds strongly through electrostatic interactions to the surface.

**Figure 5:**
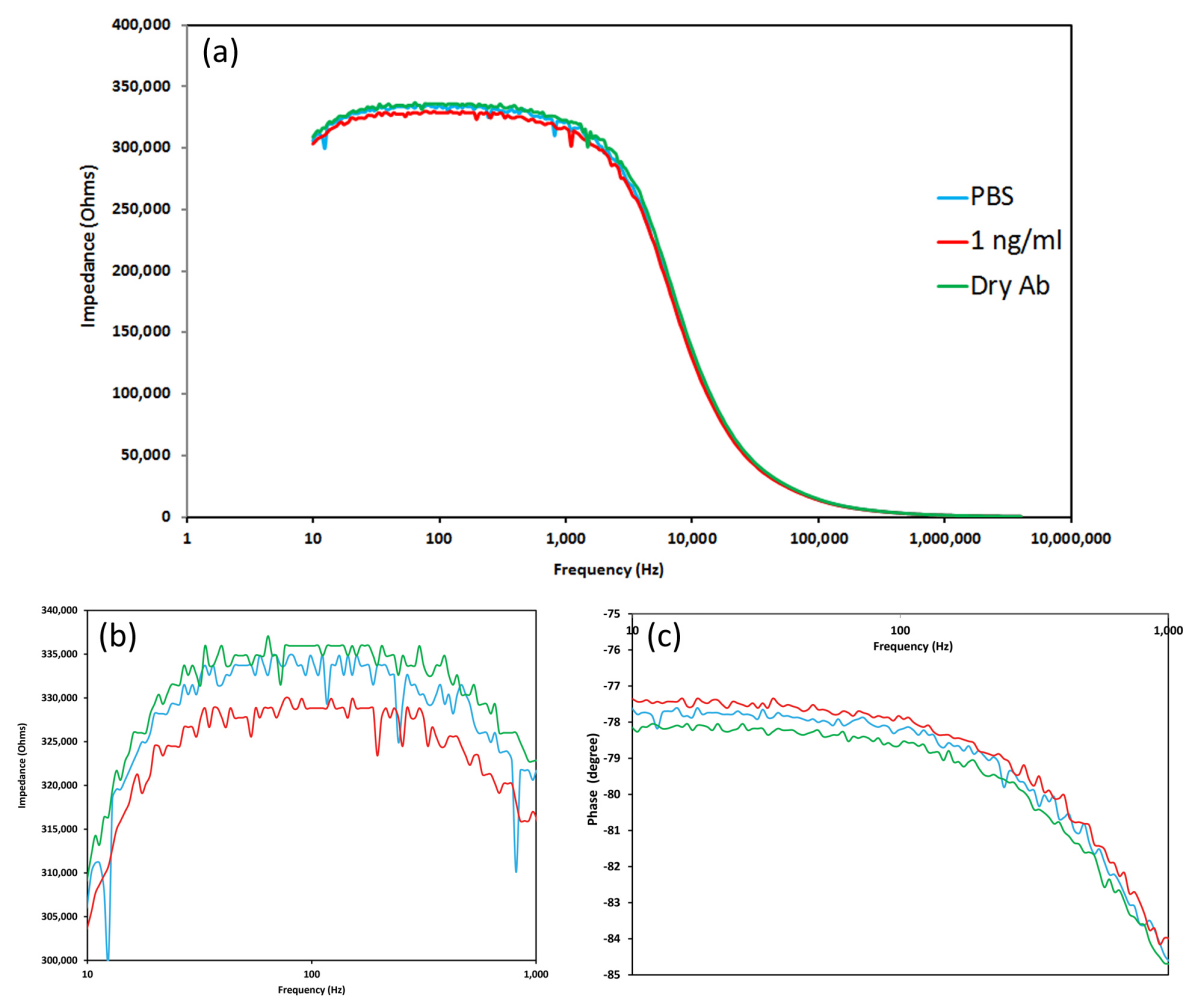
(**a**) Impedance spectroscopy (10–4 M Hz) of 1% ZnO:CuO (1:2) of 200 ng dry antibody, adding PBS and 1 ng/mL CRP for 10 min (**b**) magnified impedance spectroscopy (10–1000 Hz) and (**c**) phase plot (10–1000 Hz).

The buffer solution containing CRP, placed on the biosensor system is equivalent to be a series resistance containing salt ions with pH 7.40 and biomolecules of CRP. CRP is dominated by negative charge (pI of CRP is ∼5.45). At a fixed frequency of 138 Hz, the increased accumulation of negative charges of antigen, binding to antibody on the nano-surfaces causes an increasing capacitance of the double layer, which causes a decrease in impedance as shown in Figure 5. Figure 5 shows the impedance of nano-surfaces decreased when PBS buffer and 1 ng/mL CRP was incubated for 10 min on a 1% ZnO:CuO (1:2) nano-surface, relative to the spectra obtained from 200 ng dry anti-CRP antibody.

This also shows the large changes in impedance at low frequencies due to the electrical double layer. The phase angle value in Figure 5 also decreased when adding higher concentration of CRP onto the surface. Consequently, when binding to antibodies on the ZnO–CuO nano-surfaces, there is an increase in the overall negative charge with increasing CRP loading and a decrease in the absolute impedance and phase value.

**Figure 6:**
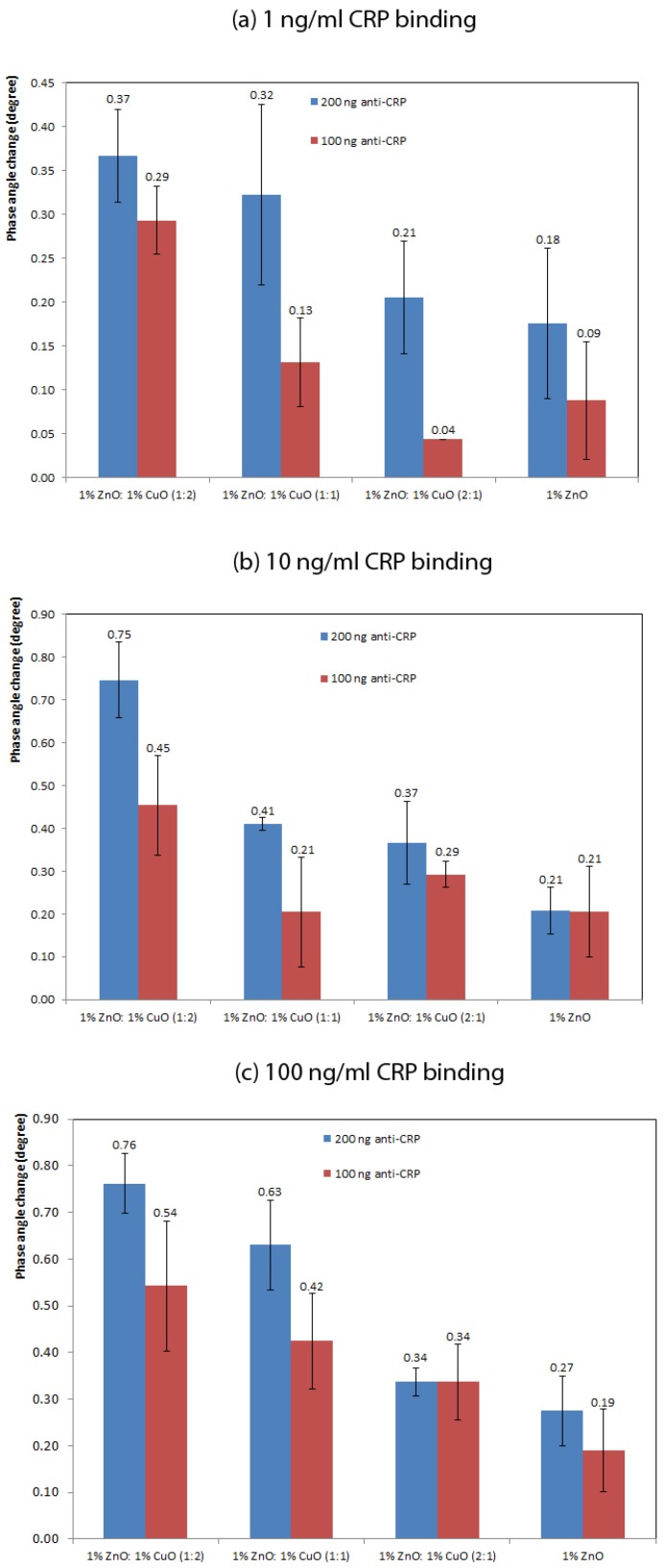
Comparisons of anti-CRP loading (100 ng and 200 ng) on different surfaces at the frequency of 138 Hz (**a**) for the detection of 1 ng/mL CRP (n = 3) with standard error bars; (**b**) for the detection of 10 ng/mL CRP (n = 3) with standard error bars; (**c**) for the detection of 100 ng/mL CRP (n = 3) with standard error bars.

Figure 6 shows the effect of adding increasing amounts of CRP to the sensor surface coated with antibody (anti-CRP) with loadings of 100 ng and 200 ng, fabricated from a pure ZnO nano-surface and from ZnO–CuO nano-surfaces with volume ratios of 1:1, 1:2 and 2:1 of ZnO:CuO. There were no results for pure CuO nano-surfaces or surface with higher ratios of CuO because the surfaces were fragile and easily broke away from the underlying substrate.

Figure 6 shows the phase change for 1, 10 and 100 ng/mL of CRP respectively (n = 3), together with standard error bars. From these Figures it can be seen that the biosensors fabricated using 200 ng antibody generated larger output signals compared with the biosensors fabricated using 100 ng antibody. This is an expected observation as there are more binding sites available for antigen capture resulting in greater protein loading on the surface and thus a greater signal generation with 200 ng of antibody. The specificity of a sensor surfaces composed of 1% ZnO (n = 2) with 200 ng anti-CRP antibody was assessed by comparing the sensor response to 125 ng/mL cortisol and 100 ng/mL CRP. The cortisol gave a phase change of 0.04 whereas the CRP samples gave a reading of 0.32 (*p* ≤ 0.05).

Interestingly, Figure 6 illustrates that increasing the relative amount of CuO in the nano-surface increases the signal output for all concentrations of CRP. Across all concentrations of CRP the biosensors with 67% CuO (1:2 ZnO:CuO) gave the greatest change in output signal, whereas the sensors with no CuO gave the smallest output changes. The effect of increasing the CuO content of the sensors was statistically significant at higher CRP concentrations for the sensors fabricated with 200 ng antibody, *p* = 0.033 for 100 ng/mL CRP and *p* = 0.029 for 10 ng/mL CRP. For 1 ng/mL CRP, there was not a significant effect of the CuO content, *p* = 0.275. This is likely to be due to the small changes in signal and the fact there is little difference in the signal output between sensors with 33% CuO and those with no CuO. The explanation for the improvement in the output with increased CuO could relate to an increase in the efficiency of charge accumulation on the nano-surface as a result of CRP binding to the antibody and a decrease in the resistance to the transient currents within the ZnO–CuO composite. Batra et al. demonstrated that ZnO–CuO composites have better conductivity than ZnO only which is attributed to the low band gap energy of CuO (1.2 eV) and the participation of holes within the semi-conductor structure [11]. The higher resistance of ZnO–CuO sensor indicates the formation of a p-n junction depleting electrons from ZnO layer more effectively than oxygen adsorption [4]. Wang et al. [5] compared ZnO–CuO composite nanotube sensor with a ZnO sensor, the response of both increased with increasing of gas concentrations. The initial resistance of ZnO-CuO composite at 300 ${}^{\circ }$C showed higher resistance because of the high resistance of CuO phase in the composite [5]. They also found that the sensitivity of ZnO–CuO composite is over three times greater than ZnO with both faster response and recovery times because p-type CuO forms a heterocontact interface with the ZnO [5]. The phase change is based on the relative charge accumulation on different nano-surfaces. Greater levels of CuO will increase the resistance and the sensitivity to charge accumulation. In all cases but one, increasing the concentration of CRP added to the biosensors resulted in an increase in output signal, the maximum output occurring for 1:2 ZnO:CuO at 100 ng/mL CRP with an antibody loading of 200 ng.

### 3.5. Sensor Response to CRP

**Figure 7:**
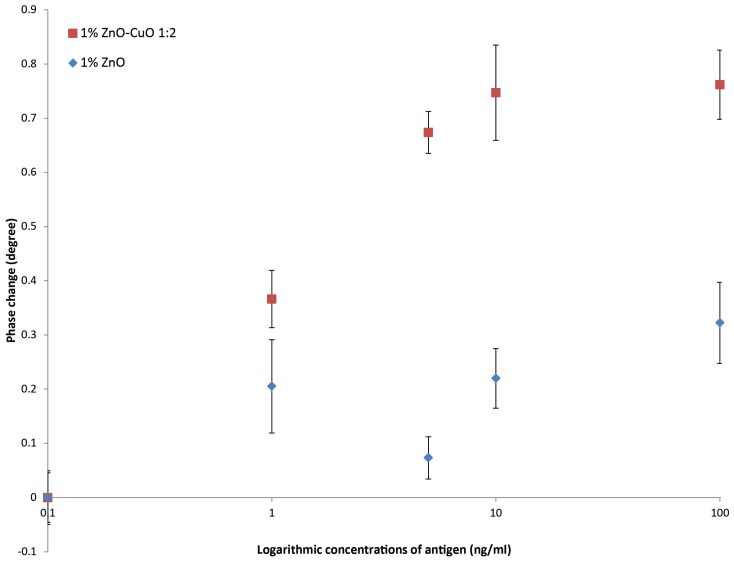
Sensor response to increasing concentrations of CRP added to nano-surfaces with the ratio of 1:2 ZnO-CuO and pure ZnO nano-surfaces (n = 3) at the frequency of 138 Hz with 200 ng capture antibody-standard error bars are shown.

Figure 7 shows a comparison of the dose response curves using biosensors fabricated with 1:2 ZnO–CuO and biosensors fabricated with no CuO, each coated with 200 ng anti-CRP. The biosensor fabricated using ZnO–CuO showed a three-fold increase in signal at the higher concentrations of CRP compared with the pure ZnO. Although the ZnO only biosensor gave an increasing signal with increasing CRP concentration this was not significant. In contrast when ZnO–CuO is used there is a highly significant increase in the signal between 1 and 10 ng/mL. Above 10 ng/mL CRP the biosensor becomes saturated with no significant difference seen in the signal generated for 10 and 100 ng/mL CRP. In addition there was a significant deference between the blank and 1 ng/mL (*p* < 0.01).

The model used to test the effect of adding CuO to ZnO nano-surfaces in a biosensor was the measurement of CRP. Using an antibody loading of 200 ng and the 1:2 ZnO–CuO nano surface, 1.0 ng/mL CRP was detected in 10 min with the potential of sub-ng/mL concentrations easily detected. Our results indicate that a sensitive biosensor for measuring CRP could be developed using a ZnO–CuO nano-crystalline surface. Wang et al. described a ZnO biosensor for CRP which had a detection limit of 100 ng/mL using a quartz crystal microbalance [18] and Ibupoto et al. described the use of ZnO nanorods to measure CRP using a potentiometric technique which was able to detect 10 ng/mL CRP [19]. Borse and Srivastava described a lateral flow technology using quantum dots as the detection technology which gave a detection limit of 300 ng/mL for CRP [20].

A biosensor developed using a ZnO–CuO nano-surface for the measurement of CRP as described in this paper would be more sensitive than current point-of-care lateral flow technologies. The integration of ZnO–CuO nano-surface technology and paper based diagnostics would provide high sensitivity and a rapid measurement for point-of-care-testing.

## 4. Conclusions

ZnO–CuO composite nano-surfaces were fabricated using a colloidal dispersion technique, incorporating sonication. SEM images and AFM 3D surfaces indicated that the nano-surfaces were modified when increasing amounts of CuO were added to form ZnO–CuO nano-surfaces. The results illustrated that the smaller CuO nanoparticle flakes filled the large voids in the ZnO surface and also align themselves flat against the ZnO surface area due to electrostatic attraction, thereby reducing the overall surface area.

Although there was not a significance difference in the antibody loading on the 1:2 ZnO–CuO compared with the ZnO only nano-surface, there was a significantly higher signal produced from the biosensor fabricated with 1:2 ZnO–CuO compared with the biosensor fabricated with ZnO only. This suggests that the electrical interaction between ZnO and CuO nano-structures plays an important part in the generation of the enhanced signal. This supposition is supported by the observations that increasing the amount of CuO in the nano-surface increases the sensor output.

We believe that this is the first time that the enhancing effect of CuO as a component of a nano-structured surface of a biosensor has been described emphasising the role of nano-material play in the development of future biosensor technologies. In addition to enhance sensitivity the biosensor is easily fabricated and would be well suited to development of point-of-care devices for the health care industry. The ability to monitor health conditions at home or to rapidly diagnose inflammatory responses or infections is driven by the development of technologies such as described in this paper, leading to cost reductions in state health care.

## Figures and Tables

**Table 1:** Image surface area comparisons of nano-surfaces analysed by NanoScope Analysis software.

Different Types of Mixed Nano-Surfaces	Image Surface Area (μm${}^{2}$) of 3 μm Scan Size	Mean Roughness (Ra) (nm)
1% ZnO	14.6	109
1% CuO	14.1	120
1% ZnO:1% CuO (1:2)	10.3	65.7

**Table 2:** Absorbance values associated with anti-mouse Ig-HRP on various nano-surfaces after washing (n = 2).

ZnO-CuO Nano-Surfaces	Average Absorbance	95% Confidence Interval
CuO	0.579	0.5692–0.5888
1:2 ZnO:CuO	0.5955	0.42204–0.76896
1:1 ZnO:CuO	0.416	0.38268–0.44932
2:1 ZnO:CuO	0.656	0.65208–0.65992
ZnO	0.5545	0.51432–0.59468
